# CAPL: an efficient association software package using family and case-control data and accounting for population stratification

**DOI:** 10.1186/1471-2105-12-201

**Published:** 2011-05-25

**Authors:** Ren-Hua Chung, Michael A Schmidt, Eden R Martin

**Affiliations:** 1 Center for Genetic Epidemiology and Statistical Genetics, John P. Hussman Institute for Human Genomics, University of Miami Miller School of Medicine, Miami, FL, USA

## Abstract

**Background:**

With many genome-wide association study (GWAS) datasets available, it is critical that we have statistical tools that are both flexible to accommodate different study designs and fast. We recently proposed the combined APL (CAPL) method, which can use family and case-control datasets and can account for population stratification in the data. Because computationally intensive algorithms are used in CAPL, implementing CAPL with efficient parallel algorithms is essential.

**Results:**

We used a hybrid of open message passing interface (open MPI) and POSIX threads to parallelize CAPL, which enable the program to operate in a cluster environment. We used simulations to demonstrate that the parallel implementation of CAPL can analyze a large GWAS dataset in a reasonable time frame when a parallel computing resource is available.

**Conclusions:**

As many GWAS datasets based on both family and case-control designs are available, a flexible and efficient tool such as CAPL will be very helpful to combine the datasets to greatly increase statistical power and finish the analysis in a reasonable time frame.

## Background

Family-based and case-control association designs have been used in many genome-wide association studies (GWAS). For GWAS where ~1 million markers are tested, the major challenge is sorting out true positives from the many false positives. Many GWAS datasets have been deposited into public databases such as the database of Genotypes and Phenotypes (dbGaP). Also the Welcome Trust Case Control Consortium (WTCCC) provides a large number of case-control samples for public analysis [1]. These resources provide the crucial opportunity to increase power by combining datasets. However, this requires flexible analytic methods that can accommodate diverse study designs (e.g., family and case-control).

Current available software for combining case-control and family data all have restrictions. Most of them such as SCOUT [2], CHRR [3] and UNPHASED [4] require sampling a homogeneous population, which may not be a reasonable assumption for data from a large consortium. FamCC [5] can account for population stratification and uses nuclear families with arbitrary number of siblings but requires parental genotype data, which are often unavailable for late-onset diseases. To overcome these restrictions, we have developed the Combined APL test (CAPL) [6], which is a novel and powerful statistical test that can accommodate family and case-control datasets and can account for population stratification using a clustering algorithm.

CAPL is an extension of the family-based Association in the Presence of Linkage (APL) test [7], which compares the difference between the observed number of alleles in affected siblings and its expected value, conditional on parental genotypes, under the null hypothesis of no linkage or no association. CAPL can use nuclear families with one or more affected sibs and can infer missing parental genotypes properly in the presence of linkage by accounting for the identity-by-descent (IBD) parameters. Unrelated cases and controls in CAPL are treated as families with one sibling and two missing parents so that they can be integrated into the family-based framework. Ward's clustering algorithm is used in CAPL to identify subpopulations and parental mating-type probabilities are calculated conditional on the subpopulation information. The EM algorithm is used to estimate the allele frequencies, IBD parameters and probabilities of origin in the presence of population substructure. A bootstrap approach is used in CAPL to estimate the variance for the CAPL statistic [8]. For each bootstrap replicate, samples are resampled with replacement and the EM algorithm is performed. The clustering algorithm is also included in the bootstrap procedure to account for the variation from clustering. CAPL has been shown to have correct type I error rates and has more power than other association tests that combine case-control and family data such as UNPHASED, SCOUT, CHRR and FAMCC under various simulation scenarios [6].

Generally 20-40 EM iterations are required for the parameter estimates to converge, and 200-1000 bootstrap replicates are performed in CAPL for the variance estimate. For each bootstrap replicate, the EM algorithm is performed. Therefore, the CAPL algorithm is very computationally intensive and can be inefficient for analyzing GWAS data. The same is also true for other association methods that infer missing parental mating types based on sample allele frequencies such as UNPHASED, which relies on the quasi-Newton algorithm for maximum likelihood estimates [4]. However, because each marker in CAPL is analyzed independently, analysis of each marker can potentially be parallelized to reduce the run time.

We implemented CAPL using the POSIX threads (pthreads) and open message passing interface (open MPI) libraries that can be executed in a computer cluster environment. We used computer simulations to demonstrate that CAPL can analyze GWAS datasets within a reasonable amount of time. The CAPL software package will be a useful tool to combine existing family and case-control GWAS datasets in the presence of population stratification.

## Implementation

**Figure 1:**
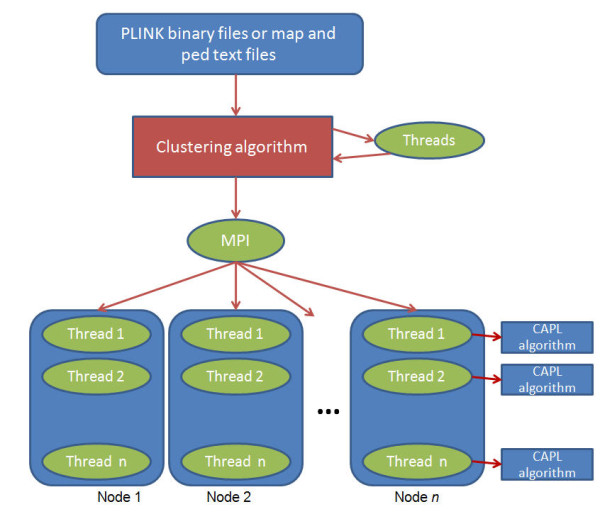
**The flowchart for the parallel CAPL algorithm**.

We used a hybrid of open MPI and pthreads to parallelize CAPL. In a cluster environment, CAPL is first executed on one node, which we denote as the master node, that manages the I/O for reading marker and individual information and writing results. The first stage in the CAPL algorithm is to perform the clustering procedure for population stratification, which requires a distance matrix. Calculating the distance matrix is time consuming because the distance is calculated for each pair of individuals over genome-wide markers. We used pthreads to parallelize the calculations on the master node. Depending on the number of computing nodes specified by the user, genotypes for markers along with the population substructure information are then distributed evenly to each node via MPI. On each node markers are analyzed independently and in parallel using pthreads with shared memory. The advantage of using pthreads instead of MPI on each node is that pthreads communicate via shared memory which is significantly faster than MPI that has to communicate via the network. Figure 1 shows the flowchart for the parallel CAPL algorithm. For a standalone machine with multiple cores, we also implemented a version of CAPL with pthreads only.

To evaluate the performance of CAPL, we used genomeSIMLA [9] to simulate GWAS datasets. We downloaded the configuration file for simulating GWAS data based on Affymetrix 550 k chip from the genomeSIMLA website. We simulated two scenarios. For the first scenario, we simulated one population based on the 600th generation from genomeSIMLA. A total of 2000 cases, 2000 controls and 1500 families were simulated, which are similar to the sample sizes of recent GWAS studies [10,11]. A total of 750 families are triads and 750 families are multiplex (parents and two affected siblings). For the second scenario, we simulated 1000 cases, 1000 controls and 750 trio families from one population (based on the 600th generation in genomeSIMLA) and 1000 cases, 1000 controls and 750 multiplex families from another population (based on the 750th generation). The jobs were distributed across 6 nodes, each with 8 Xeon 2.6 GHz cores and 16 GB of memory. We also performed the jobs on 50 nodes, each with 8 Xeon 2.6 GHz cores and 16 GB of memory.

We then evaluated the performance of CAPL on a single node for the two scenarios. Because the simulated datasets are large, we compiled a demonstration version of CAPL by fixing the number of bootstrap iterations to be 50 and the number of EM iterations to be 15 to reduce the run time. This demonstration version is for comparing performance only, as reducing the numbers of bootstrap and EM iterations may cause biased estimates of parameters.

## Results

The run time for scenario 1 was 2 days 2 hours and 43 minutes and the run time for scenario 2 was 3 days 6 hours and 11 minutes using 6 nodes (48 parallel threads). The run time for scenario 1 was 7 hours and 18 minutes and the run time for scenario 2 was 18 hours and 14 minutes using 50 nodes (400 parallel threads). Scenario 2 had longer run time since more parameters were estimated for population substructure analysis [6]. We can see that in this example consisting of 9250 samples and half million markers, CAPL can complete analysis in a reasonable time frame with the estimates of parental mating types, IBD and population substructure parameters when sufficient parallel computing resource is available.

For the demonstration version of CAPL (with restricted 50 bootstrap and 15 EM iterations) using 1 node (8 parallel threads), the run time for scenario 1 was 1 day 7 hours and 8 minutes and the run time for scenario 2 was 1 day 19 hours and 45 minutes. Without the restrictions on the bootstrap and EM iterations, CAPL averaged about 250 bootstrap iterations and about 40 EM iterations across markers in the simulated samples. Therefore, the total iterations (250 bootstrap × 40 EM) required for CAPL to have unbiased estimates of parameters is about 13.3 times the number of restricted iterations (50 bootstrap × 15 EM). In practice, we expect that CAPL using 1 node would require more than 17 days for similar sample size with scenario 1 and more than 24 days for scenario 2. The results demonstrate the importance of implementing CAPL with MPI and pthreads in a distributed system.

## Conclusions

CAPL is implemented with a hybrid of open MPI and pthreads, which can be performed using a computer cluster with shared memory. We also provide a version of CAPL with pthreads only, so that the program can be performed on a standalone machine with pthreads. Without the parallelization, CAPL may require months to complete a GWAS analysis using one processor. The situation is the same for other computationally intensive association software such as UNPHASED. To speed analysis, users may divide the input file into subsets of markers and manually run the subsets of markers on different machines. This is not ideal because extra storage is needed for the subsets of files. Moreover, reducing the number of markers in a subset may cause the loss of information about population substructure for the clustering algorithm in CAPL. Therefore, the parallelization for CAPL is essential for GWAS.

In conclusion, we developed the efficient software package CAPL based on open MPI and pthreads, which is a powerful association test that can accommodate case-control and family data and account for population stratification. As many GWAS datasets based on both family and case-control designs are available, a flexible and efficient tool such as CAPL will be very helpful to combine the datasets to greatly increase statistical power and finish the analysis in a reasonable time frame. Moreover, population stratification is properly accounted for in CAPL so that datasets from different populations can be jointly analyzed.

## Software Configuration

We compiled the CAPL code for the standalone version, which can be executed on a single machine with multiple cores, and the cluster version, which can be executed in a cluster environment. We distribute binaries for the standalone version (for Windows, Mac OS, and Linux) and a binary for the cluster version (for Linux). When executing the Windows binary, the pthreads library (pthreadVC2.dll) needs to be in the same directory as the binary. For Linux and Mac OS users, we strongly encourage the users to compile the code on their machines, as the configurations of dynamic links to libraries may vary on different machines.

In addition to binaries, we provide source code with sets of makefiles for the standalone and cluster versions. The standalone version can be compiled with GNU's g++ 4.1.2 compiler or later versions. The cluster version can be compiled with mpiCC. We also provide makefiles based on Intel compilers for Intel hardware users. Based on our experiments, CAPL has better performance on the Intel hardware when it was compiled with the Intel compiler. We provide examples of the submission scripts for the cluster based on the commonly used LSF and MOAB job schedulers. Users may need to work with their cluster administrators to set up the parameters in the submission scripts properly. For example, to achieve the optimal performance for CAPL, for each computing node, the whole node needs to be reserved for the job and the number of threads to run the job on each node needs to be specified correctly in the submission script.

A control file is also required for the user to run CAPL. The user needs to specify the input file format and path. Currently the PLINK [12] binary file (bim, bed and fam files) or the text file (map and ped files) formats are accepted by CAPL. Other important parameters in the control file are the number of threads, number of nodes, number of subpopulations for the clustering algorithm, and the name of the result file. Note that the numbers of threads and nodes need to be the same as the parameters specified in the submission script for the cluster. More details about the configurations of the CAPL software can be found in the software documentation.

## Availability and requirements

**Project name: **CAPL

**Project home page: **http://www.mihg.org/software_download/download_reg.php?software=CAPL

**Operating system(s): **Linux (both standalone and cluster versions), Windows (standalone version), and Mac OS (standalone version).

**Programming language: **C++

**License: **GNU GPL

**Any restrictions to use by non-academics: **None

**Download statistics: **16 unique outside users since the launch of the CAPL download site

## Authors' contributions

All co-authors contributed to writing the manuscript. RHC was the primary author on the manuscript, developed the statistical methods and the design for their implementation. MAS contributed to the implementation of the software. ERM provided input to study design and interpretation of data. All authors read and approved the final manuscript.
